# SVCT2 vitamin C transporter expression in progenitor cells of the postnatal neurogenic niche

**DOI:** 10.3389/fncel.2013.00119

**Published:** 2013-08-13

**Authors:** Patricia Pastor, Pedro Cisternas, Katterine Salazar, Carmen Silva-Alvarez, Karina Oyarce, Nery Jara, Francisca Espinoza, Agustín D. Martínez, Francisco Nualart

**Affiliations:** ^1^Laboratory of Neurobiology and Stem Cells, Department of Cellular Biology, Center for Advanced Microscopy CMA BIOBIO, University of ConcepciónConcepción, Chile; ^2^Centro Interdisciplinario de Neurociencia de Valparaíso, Facultad de Ciencias, Universidad de ValparaísoValparaíso, Chile

**Keywords:** SVCT2, vitamin C, brain, niche, stem cells, progenitor, ependymal cells

## Abstract

Known as a critical antioxidant, recent studies suggest that vitamin C plays an important role in stem cell generation, proliferation and differentiation. Vitamin C also enhances neural differentiation during cerebral development, a function that has not been studied in brain precursor cells. We observed that the rat neurogenic niche is structurally organized at day 15 of postnatal development, and proliferation and neural differentiation increase at day 21. In the human brain, a similar subventricular niche was observed at 1-month of postnatal development. Using immunohistochemistry, sodium-vitamin C cotransporter 2 (SVCT2) expression was detected in the subventricular zone (SVZ) and rostral migratory stream (RMS). Low co-distribution of SVCT2 and βIII-tubulin in neuroblasts or type-A cells was detected, and minimal co-localization of SVCT2 and GFAP in type-B or precursor cells was observed. Similar results were obtained in the human neurogenic niche. However, BrdU-positive cells also expressed SVCT2, suggesting a role of vitamin C in neural progenitor proliferation. Primary neurospheres prepared from rat brain and the P19 teratocarcinoma cell line, which forms neurospheres *in vitro*, were used to analyze the effect of vitamin C in neural stem cells. Both cell types expressed functional SVCT2 *in vitro*, and ascorbic acid (AA) induced their neural differentiation, increased βIII-tubulin and SVCT2 expression, and amplified vitamin C uptake.

## Introduction

Active neurogenesis occurs within the anterior wall of the lateral ventricle in the adult mammalian brain (Lois and Alvarez-Buylla, 1993; Doetsch et al., 1999). Neurogenic precursors have also been found in the human brain, specifically located in the periventricular region before 18 months of age (Johansson et al., 1999; Nunes et al., 2003; Sanai et al., 2011; Bergmann et al., 2012). The formation of new neurons, which are βIII-tubulin-positive, occurs in restricted, organized compartments termed neurogenic niches (Doetsch et al., 1997, 1999; Alvarez-Buylla and Garcia-Verdugo, 2002; Conover and Notti, 2008; Mirzadeh et al., 2008; Nualart et al., 2012). The neuroblasts formed in this region migrate tangentially in chains throughout the rostral migratory stream (RMS), where the presence of neurogenic progenitors and astrocytes has also been described (Doetsch and Alvarez-Buylla, 1996). The neuroblasts present in the RMS reach the olfactory bulb, where they differentiate into interneurons (Lois et al., 1996; Alvarez-Buylla and Garcia-Verdugo, 2002; Lledo et al., 2008). Ultrastructural, immunohistochemistry, and proliferation analyses of the cytoarchitecture of the neurogenic niche (Doetsch et al., 1997) have revealed the presence of four cell types. B-type cells or astrocytes (GFAP- and nestin-positive) are preferentially located in the subventricular zone (SVZ) and are precursor cells. C-type cells are intermediate transient neuronal cells (nIPC) that proliferate rapidly (Eisch and Mandyam, 2007; Ihrie and Alvarez-Buylla, 2008) and differentiate into neuroblasts or type-A cells (Doetsch et al., 1999; Tramontin et al., 2003; Chojnacki et al., 2009; Kriegstein and Alvarez-Buylla, 2009; Nualart et al., 2012). E-type cells, which are cube-shaped and multiciliated, are ependymocytes. B-type cells are found in the ependymal layer, projecting cilium to the ventricular lumen, similar to what has been described in the radial glia (Tramontin et al., 2003; Spassky et al., 2005); they also have a close relationship with blood vessels (Mirzadeh et al., 2008). Precursor cells reactive to GFAP have been identified in the SVZ of the human brain (Roy et al., 2000; Gibbons and Dragunow, 2010), and some are in direct contact with the cerebrospinal fluid (CSF) (Sanai et al., 2004, 2011; Quinones-Hinojosa et al., 2006).

Vitamin C, which is present in high concentrations in the CSF (Spector and Lorenzo, 1974; Kratzing et al., 1985), may be important in postnatal neural differentiation. An important role for vitamin C in embryonic cerebral development and in the differentiation of dopaminergic and serotoninergic neurons has been described (Lee et al., 2000; Yan et al., 2001). Embryonic precursors supplemented with vitamin C show an increase in neural and glial markers (Lee et al., 2003). Recently, Esteban et al. (2010) found that vitamin C favored the generation of induced pluripotent stem cells (iPS) (Esteban et al., 2010). Furthermore, cells grown *in vitro* in the presence of vitamin C expressed two histone demethylases, Jhdm1a and Jhdm1b (Wang et al., 2011), which are required for iPS cell production. Together, these results suggest that vitamin C is able to positively regulate stem cell generation and proliferation.

The intracellular incorporation of ascorbic acid (AA) by neurons is carried out by SVCT2, the sodium and AA co-transporter (Daruwala et al., 1999; Castro et al., 2001; Hediger, 2002; Harrison and May, 2009; Nualart et al., 2012). This protein is formed by 12 transmembrane domains, with a molecular mass of ~75 KDa (García et al., 2005). In the CNS, SVCT2 is expressed primarily in neurons of the cerebral cortex, hippocampus, and hypothalamus (Tsukaguchi et al., 1999; García et al., 2005); its expression has also been described in microglia (Mun et al., 2006) and tanycytes of the hypothalamus (García et al., 2005). In addition, functional SVCT2 was observed in cultures of embryonic rat cortical neurons (Castro et al., 2001; Astuya et al., 2005). Recently, SVCT2 mRNA expression was detected in radial glial cells of the fetal rat brain (Caprile et al., 2009). Moreover, SVCT2 knockout mice die at birth due to respiratory defects and cerebral hemorrhaging; low levels of AA in various tissues were also noted in SVCT2-null mice (Sotiriou et al., 2002). These data suggest that SVCT2 and vitamin C are important for normal nervous system development and neuronal maturation. The neurogenic niche stem cells are in contact with the CSF, which has high a concentration of vitamin C. Therefore, vitamin C may be a factor involved in stem cell differentiation; however, studies regarding the expression and distribution of the vitamin C transporter, SVCT2, in neural stem cells of the postnatal brain neurogenic niche and the effect of vitamin C on neuronal differentiation of stem cells from the periventricular areas of the brain have not been performed.

In this study, the expression of SVCT2 at the initial stages of differentiation of the ventricular neurogenic niche was analyzed in the rat brain. In addition, the distribution of SVCT2 in the human ventricular wall at 1 month postnatal development was assessed. Using P19 cells (an *in vitro* progenitor cell line with active proliferation) and primary neurospheres isolated from rat brain, SVCT2 expression and the effects of vitamin C on neural differentiation were determined.

## Materials and methods

### Animals

Adult Sprague–Dawley rats and animals at 15–21 days postnatal development were used throughout the experiments. Animals were maintained in a 12 h light/dark cycle with food and water *ad libitum*. The handling of the animals was performed in agreement with the “Manual de Normas de Bioseguridad” (Comición Nacional de Ciencia y Tecnología, CONICYT), and the procedures were described in the publication entitled, “*Guide for Care and Use of Laboratory Animals”* (National Academy of Science, 2011; http://grants.nih.gov/grants/olaw/Guide-for-the-care-and-use-of-laboratory-animals.pdf). One month postnatal human brain tissue samples were obtained from archived samples previously fixed in 4% paraformaldehyde from the Department of Pathological Anatomy at Concepcion University. The samples were obtained in accordance with the accepted standards of the ethics committee on the use of human specimens and after informed consent was obtained from all patients.

### Immunohistochemistry and confocal microscopy

Rat brain tissue samples were fixed in formalin at 10% v/v or in Bouin solution and embedded in paraffin after which 7-μm saggital sections were obtained. For the immunohistochemical analysis, the deparaffinized samples were incubated for 15 min in absolute methanol with 3% v/v H_2_O_2_. The sections were incubated with the following primary antibodies diluted in Tris-phosphate buffer and 1% bovine serum albumin: anti-PCNA (1:100 DAKO, Carpinteria, CA, USA); anti-Nestin (1:25 Amersham Pharmacia Bitech., Pittsburgh, PA, USA); anti-βIII-tubulin (1:500, Promega, Madison, WI, USA); anti-GFAP (1:200, DAKO); anti-PSA-NCAM (1:25 Hybridoma Bank, Iowa. IA, USA); anti-S100A (1-200, DAKO); and anti-SVCT2 (G19; 1:50, Santa Cruz Biotechnology, Sta. Cruz, CA, USA). The samples were then incubated with the appropriate secondary antibody conjugated to horse radish peroxidase (HRP), including HRP-conjugated goat anti-IgG, HRP-conjugated rat anti-IgG, and HRP-conjugated rabbit anti-IgG (ImmunoPure; PIERCE Biotechnology, Rockford, IL, USA). The enzymatic activity of the peroxidase was revealed with diaminobenzidine and H_2_O_2_. To perform inmunofluorescence analysis, secondary antibodies conjugated to different fluorophores, including Cy2-conjugated goat anti-IgG, Cy2- or Cy3-conjugated rat anti-IgG; and Cy3- or Cy5-conjugated rabbit anti-IgG (Jackson Immuno Research, Pennsylvania, USA), were used. The images (512 × 512 × 8 bits or 1024 × 1024 × 8 bits) were obtained using a confocal microscope.

### *In situ* hybridization

A PCR product of 620 bp, which was obtained from pcDNA3-hSVCT2 that was subcloned into pCR-4-Blunt-TOPO (Clontech, Palo Alto, CA, USA), was used to generate sense and antisense digoxigenin-labeled riboprobes. RNA probes were labeled with digoxigenin-UTP by *in vitro* transcription with SP6 or T7 RNA polymerase following the manufacturer's instructions (Boehringer Mannheim, Mannheim, Germany). *In situ* hybridization was performed on brain sections mounted on poly-L-lysine-coated glass slides. The sections were baked at 60°C for 1 h, deparaffinized in xylene, and rehydrated in graded ethanol. Following proteinase K treatment (5 min at 37°C in PBS, 1 mg/ml), the tissue sections were fixed with 4% paraformaldehyde at 4°C for 5 min, washed in cold PBS, and then acetylated with 0.1 M triethanolamine-HCl (pH 8.0) and 0.25% acetic anhydride at room temperature for 10 min. After a brief wash, the sections were incubated in pre-hybridization solution (Boehringer Mannheim) at 37°C for 30 min, and then 25 μl of hybridization mix [50% formamide, 0.6 M NaCl, 10 mM Tris-HCl (pH 7.5), 1 mM ethylenediaminetetraacetic acid (EDTA), 1 × Denhart's solution, 10% polyethylene glycol 8000, 10 mM DL-dithiothreitol (DTT), 500 mg yeast tRNA/ml, 50 mg/ml heparin, 5.0 mg/ml DNA carrier, and 1:20–1:100 dilutions of riboprobe) were added to each slide. The slides were covered with glass coverslips and placed in a humidified chamber at 42°C overnight. The slides were washed at 37°C for 30 min each in 2 × SSC, 1 × SSC, and 0.3 × SSC. Visualization of digoxigenin was performed by incubation with a monoclonal antibody coupled to alkaline phosphatase (anti-digoxigenin alkaline phosphatase Fab fragments diluted 1:500; Boehringer Mannheim) at room temperature for 2 h. Nitroblue tetrazolium chloride and 5-bromo-4-chloro-3-indolyl-phosphate (Boehringer Mannheim) were used as substrates for the alkaline phosphatase. Controls included use of the sense riboprobe and omission of the probe.

### *In vivo* BrdU labeling

Five intraperitonial injections of BrdU at a concentration of 40 mg/kg were administered to animals at 19 days postnatal development. Samples were obtained at 21 days postnatal development and fixed in Bouin, and the sections were obtained as previously described. Briefly, after denaturation of the DNA with 2N HCl for 30 min at 37°C, the sections were incubated with 5% BSA for 30 min and anti-BrdU (1:1000, Amersham Pharmacia Biotech) overnight. Detection was made by incubation with rat peroxidase-labeled anti-IgG or rat Cy3-labeled anti-IgG.

### Cell culture

The embryo-derived teratocarcinoma cell line, P19 (McBurney, 1993), was cultured in Minimum Essential Medium Eagle (MEM) (Gibco BRL, Grand Island, NY, USA) supplemented with 5% fetal bovine serum (FBS), penicillin, and streptomycin. The neurospheres were generated in pretri dishes (10 cm in diameter) seeded with 1 × 10^6^ cells maintained in culture for 4 days in (1) MEM (control), (2) MEM supplemented with 200 or 400 μM AA or (3) cultured in neurobasal-B27 (Gibco-BRL).

For neurosphere preparation, adult Sprague–Dawley rats were sacrificed by cervical dislocation (*n* = 6 per preparation), and their brains were removed. The lateral walls of the lateral ventricle were dissected and collected in DMEM-F12 (GIBCO). The cells were dissociated using a Pasteur pipette in NeuroCult NS-A (Stem Cells Technologies Inc), and cells were collected by centrifugation at 100 × g for 5 min. The cell pellets were resuspended in NeuroCult NS-A, supplemented with 20 ng/ml EGF, 10 ng/ml bFGF, 2 μg/ml heparine and proliferation supplement (Stem Cells Technologies Inc). The cells were cultured at 40,000 cells per cm^2^ on uncoated dishes in the same medium. To analyze the effects of vitamin C, neurospheres were cultured for 7 days and were treated with 200 or 400 μM AA the last 4 days. The same concentration of vitamin C was added to the cultured cells each day.

### Reverse transcription-polymerase chain reaction

Total RNA was isolated using Trizol (Invitrogen, Rockville, MD, USA). For RT-PCR, 1 μg of RNA was incubated in 10 μl reaction volume containing 10 mM Tris-HCl (pH 8.3), 50 mM KCl, 5 mM MgCl_2_, 20 U RNase inhibitor, 1 mM dNTPs, 2.5 μM of oligo d(T) primers, and 50 units of MuLV reverse transcriptase (New England Biolabs, Ipswich, MA, USA) for 10 min at 23°C followed by 30 min at 42°C and 5 min at 94°C. Parallel reactions were performed in the absence of reverse transcriptase to control for the presence of contaminant DNA. For amplification, a cDNA aliquot in a volume of 12.5 μl containing 20 mM Tris-HCl (pH 8.4), 50 mM KCl, 1.6 mM MgCl_2_, 0.4 mM dNTPs, 0.04 units of *Taq* DNA polymerase (Gibco-BRL) and 0.4 mM primers was incubated at 95°C for 5 min, 95°C for 30 s, 50°C for 30 s, and 72°C for 30 s for 35 cycles with a final extension at 72°C for 7 min. PCR products were separated by 1.2–1.5% agarose gel electrophoresis and visualized by staining with ethidium bromide. The following set of primers was used to analyze the expression of SVCT2: sense: 5′TGTTTCAGGCCAGTGCTTT 3′ and antisense: 5′GAAAGGATGGACGGCATACA 3′ (expected product of 457 bp). Additionally, the following sets of primers were used to analyze the expression of nestin and βIII-tubulin: nestin sense, 5′GGAGTCTCGCTTAGAGGTGC 3′ and antisense, 5′ CAGCAGAGTCCTGTATGTAGCC 3′ (expected product 103 bp) and βIII-tubulin sense, 5′ TTTATCTTCGGTCAGAGTGG 3′ and antisense, 5′ GAGCAGCAGTAGAAGTATGT 3′ (expected product 1500 bp).

### Western blot analysis

Membrane proteins from P19 cells or neurospheres were obtained by homogenizing the cells in 0.3 mM sucrose, 3 mM DTT, 1 mM EDTA, 1.0 mg/ml PMSF, 1 mg/ml pepstatin A, 2 mg/ml leupeptin, and 2 mg/ml aprotinin. Total membranes were collected by high-speed centrifugation. For immunoblotting, 50 mg of membrane protein was loaded in each lane and separated by polyacrylamide gel electrophoresis in the presence of sodium dodecyl sulfate, transferred to PVDF membranes (0.45 μm pore, Amersham Pharmacia Biotech., Piscataway, NJ, USA) and probed against an anti-SVCT2 (1:100) antibody or a preabsorbed antibody (1:500) (García et al., 2005). The secondary antibody was rabbit anti-goat IgG coupled to peroxidase (1:5000). The reaction was developed with enhanced chemiluminescence according to the manufacturer's instructions (Amersham Corporation, Arlington Heights, IL).

### Vitamin C uptake studies

P19 cells were carefully selected under the microscope to ensure that only plates showing uniformly growing cells were used at 200,000 cells/well. Additionally, neurospheres isolated from rat brain were also used for vitamin C uptake. The cells were incubated in buffer containing 15 mM HEPES (N-2-hydroxyethylpiperazine-N'-2-ethanesulfonic acid), 135 mM NaCl, 5 mM KCl, 1.8 mM CaCl2, 0.8 mM MgCl2 at room temperature for 30 min. Uptake assays were performed in 500 ml of incubation buffer containing 0.1–0.4 mCi of 1-^14^C-AA (Dupont NEN, Boston, MA, USA, specific activity 8.2 mCi/mmol) to a final concentration of 100 mM. Data represent means ± SD of three experiments with each analysis performed in duplicate. To analyze the effect of AA on SVCT2 expression and function, P19 cells were pre-incubated with 400 mM AA for 4 days. Initial velocity transport assays were performed after incubation with 100 mM AA with or without 135 μM sodium chloride (replaced by sodium choline) or 20 μM cytochalasin B.

### Statistical analysis

For inhibition experiments, statistical comparisons between two or more groups of data were carried out using analysis of variance (ANOVA, followed by Bonferroni post-test). *P* < 0.05 was considered to be statistically significant. The statistical software, GraphPad Instat 3.0 (GraphPad Software, Inc., La Jolla, CA 92037 USA), was used for data analysis.

## Results

### Postnatal neurogenic niche differentiation

**Figure 1:**
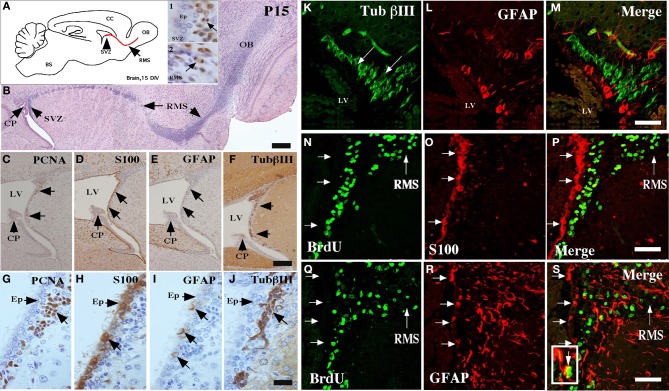
**Characterization of the neurogenic niche at 15 days postnatal development. (A)** Scheme of a sagittal section of rat brain, highlighting the anterior ventricular zone, the neurogenic niche of the subventricular zone (SVZ), and the rostral migratory stream (RMS). Insets 1 and 2: Immunohistochemical analysis with anti-PCNA (1:100) in the SVZ and the RMS, respectively. **(B)** Sagittal cut of rat brain stained with hematoxylin and eosin showing the RMS (arrows). **(C–J)** Neurogenic niche of the brain at 15 days postnatal development analyzed with anti-PCNA, anti-S100a, anti-GFAP, and βIII-tubulin. **(K–S)** Immunofluorescence and confocal microscopy analyses using antibodies to identify glial and neural cells in different states of proliferation. **(K–M)** Brain tissue analyzed with anti-βIII-tubulin and GFAP. **(N–P)** Co-localization of S100a-positive ependymal cells and BrdU-positive cells. **(Q–S)** Co-localization of GFAP-positive glial cells and BrdU-positive cells. The images are representative of three independent experiments. CP, choroid plexus; LV, lateral ventricle; OB, olfactory bulb. Scale bars in **(B)**, 250 μm; **(C–J)**, 100 μm; **(K–S)**, 50 μm.

The ventricular neurogenic niche of postnatal brains at 15 days showed a similar cellular structure to that described in the adult brain (Doetsch et al., 1997; Alvarez-Buylla and Garcia-Verdugo, 2002; Alvarez-Buylla and Lim, 2004). The ependymal cells were differentiated in the ventricular wall, and the SVZ was formed by different cell types (Figure 1). The migration of cells to the olfactory bulb, the RMS, was also assessed (Figures 1A, insets 1–2 and **B**). At 15 days, a positive reaction with anti-PCNA in the SVZ was observed (Figures 1A, inset 1, **C,G**); the ependymal cells were also labeled with anti-S100A (Figures 1D,H). Type-B precursor cells (GFAP-positive) were also detected in the SVZ (Figures 1E,I). βIII-tubulin expression was observed in neuroblasts (Figures 1F,J,K, arrows) surrounded by B-type cells reactive to GFAP (Figures 1L,M). GFAP-positive cells presented radial morphology, and their processes extended to the subventricular region (Figures 1L,M).

Mitotically active cells were next detected by *in vivo* BrdU administration. All the BrdU-positive cells were observed in the subventricular region (Figure 1N), a result that was confirmed by analysis with anti-S100, an ependymal cell marker (Figures 1O,P). Approximately, ten percentage of the BrdU-positive cells were also GFAP-positive (Figures 1Q–S). BrdU-positive cells were also detected at the RMS (Figure 1 N,Q).

### Expression of SVCT2 in the neurogenic niche and RMS

**Figure 2:**
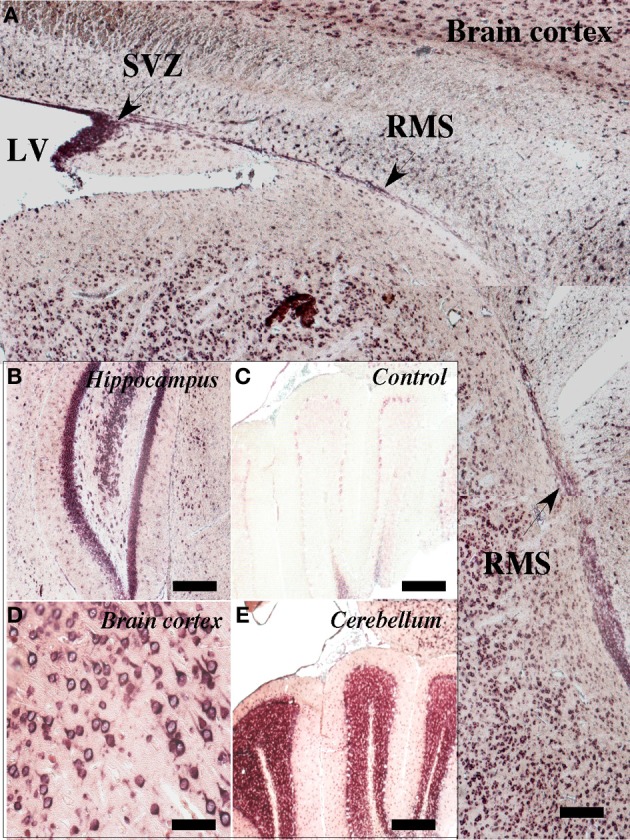
**Expression of SVCT2 mRNA in the neurogenic niche and RMS**. *in situ* hybridization analysis of a sagittal section of rat brain at 21 days postnatal development. **(A)** A positive reaction was observed in the anterior wall of the lateral ventricle as well as in the ventricular and subventricular zones. A positive reaction was also observed in the RMS (arrows). **(B,D,E)** Positive *in situ* hybridization in different regions of the CNS: hippocampus **(B)**, cerebral cortex **(D)**, cerebellum **(E)**. **(C)** Negative control using the sense probe. LV, lateral ventricle; SVZ, subventricular zone; RMS, rostral migratory stream. Scale bar, 100 μm.

Using *in situ* hybridization, SVCT2 mRNA expression was observed in the anterior ventricular zone and RMS (Figure 2A). SVCT2 mRNA expression was also located in other areas of the brain, including neurons of the hippocampus (Figure 2B), cerebral cortex (Figure 2D), and the cerebellum (Figure 2E). As a negative control, the sense probe was employed; no reaction was observed in the different cerebral areas analyzed (Figure 2C). Furthermore, a positive reaction to astrocytes was not observed; however, ependymal cells presented an intense reaction in different regions of the ventricular wall (data not shown).

**Figure 3:**
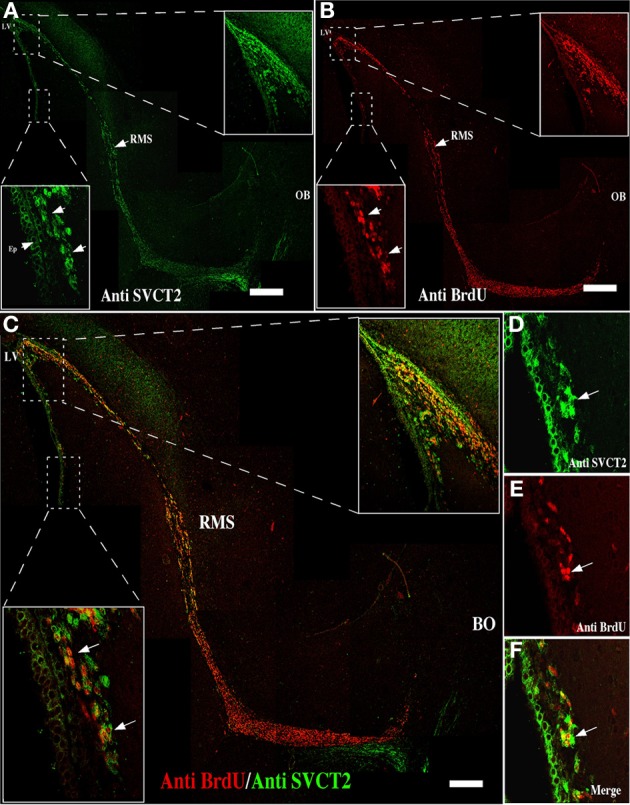
**Mitotically active cells express SVCT2**. Immunofluorescence and confocal microscopy analyses of the neurogenic niche and RMS at 21 days postnatal development, using antibodies to identify proliferative cells and the ascorbic acid transporter, SVCT2. **(A)** Sagittal section of the rat brain analyzed with anti-SVCT2. **(B)** Sagittal section of rat brain analyzed with anti-BrdU, 5 days after incorporation of BrdU *in vivo*. **(C–F)** Co-localization of SVCT2- and BrdU-positive cells in the neurogenic and RMS areas. The images are representative of three independent experiments. LV, Lateral ventricle; RMS, rostral migratory stream. Scale bars in **(A–C)**, 100 μm. Arrows point positive cells for SVCT2 and BrdU.

García et al. (2005) had demonstrated that SVCT2 mRNA expression did not directly correlate with its protein levels. Specifically, neurons presenting an intense *in situ* hybridization signal for SVCT2 showed a weak immunoreactivity with anti-SVCT2. Thus, SVCT2 protein expression was determined using immunofluorescence and confocal microscopy in the neurogenic niche, and intense immunoreactivity in the anterior ventricular area and RMS was detected (Figure 3A, insets). The positive signal was observed in the ependymal cells and different cells present in the SVZ (Figure 3A, inset and arrows). To determine whether SVCT2 was associated with proliferative cells, co-localization analysis was undertaken with BrdU-positive cells (Figure 3B, insets). Most of the BrdU-positive cells were also SVCT2-positive (Figure 3C, insets), and a high percentage of these cells were localized in the SVZ (Figures 3C, insets and D–F). The number of BrdU/SVCT2-positive cells was next quantified in two rat brains; of 494 BrdU-positive cells in the neurogenic niche, 95.1% were also SVCT2-positive.

**Figure 4:**
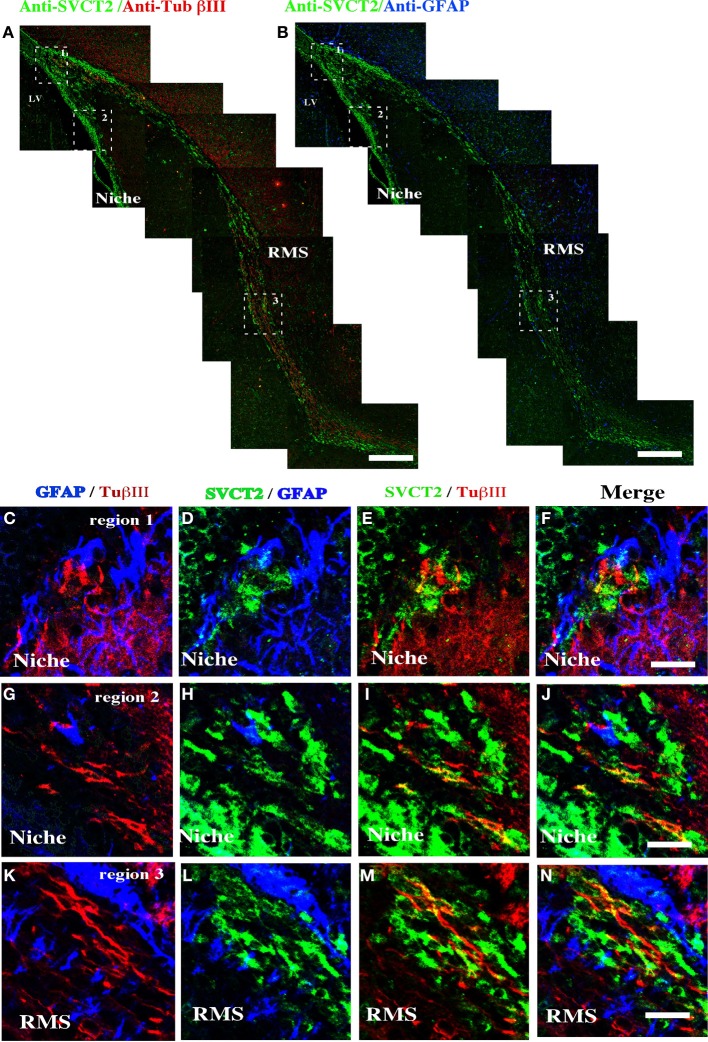
**Distribution of SVCT2, GFAP, and βIII-tubulin in the cells of the ventricular area and the RMS**. Immunofluorescence and confocal microscopy analyses of the ventricular neurogenic niche after 21 days postnatal development using antibodies to identify glial cells, neurons and SVCT2. **(A)** Sagittal section of rat brain analyzed with anti-SVCT2 and anti-βIII-tubulin. **(B)** Sagittal section of rat brain analyzed with anti-SVCT2 and anti-GFAP. **(C–P)** Co-localization of glial and neural cells with anti-SVCT2 (green), anti-GFAP (blue), and anti-βIII-tubulin in the dorsal **(C–F)** and ventral neurogenic niche **(G–J)**. **(K–N)** Co-localization of glial and neural cells in the RMS. The images are representative of three independent experiments. Ep, ependymal cells. LV, lateral ventricle. Scale bars in **(A–B)**, 100 μm; **(C–N)**, 20 μm.

**Figure 5:**
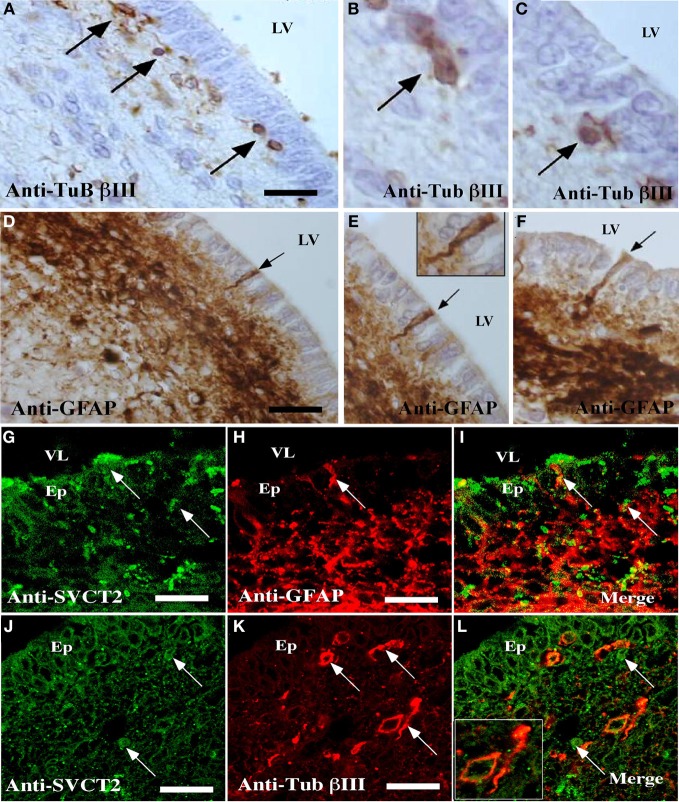
**Detection of cells reactive to GFAP, βIII-tubulin, and SVCT2 in the ventricular wall of the postnatal human brain. (A–F)** Immunohistochemical analysis using antibodies to identify glial and neural cells. **(A–C)** Sagittal section of the ventricular wall of the human brain at 1 month postnatal development analyzed with antibodies specific for βIII-tubulin **(A–C)** or anti-GFAP **(D–F)**. **(G–L)** Immunofluorescence and confocal microscopy analyses of the human ventricular neurogenic niche, using antibodies to identify glial and neural cells and the SVCT2. Sagittal section of the ventricular wall analyzed with anti-GFAP and anti-SVCT2 **(G–I)** or anti-βIII-tubulin and anti-SVCT2 **(J–L)**. The images are representative of three independent experiments. Ep, ependymal cells; LV, lateral ventricle. Scale bars in **(A–K)**, 20μm. Arrows point positive cells for the respective antibodies.

To determine the cell types within the neurogenic niche that express SVCT2, immunofluorescence analysis with triple labeling was undertaken (Figure 4). At 21-days postnatal development, SVCT2 did not co-localize with βIII-tubulin (Figure 4A) or with GFAP (Figure 5B). High power imaging in different regions of the neurogenic niche and in the RMS confirmed these observations (Figures 4C–N). SVCT2 reactions were observed in ependymal cells and cells located in the SVZ, again without co-localizing with GFAP (Figures 4D,H) and with low co-localization with βIII-tubulin (Figures 4E,I). A similar result was found in the RMS (Figures 4K–N), suggesting that SVCT2 is present in highly proliferative intermediate progenitors (C type cell or nIPC).

Analysis of the ventricular wall of a human brain at 1 month postnatal development was next performed. The presence of βIII-tubulin-positive neuroblasts in the SVZ was observed (Figure 5). These cells had varying morphologies; some were spherical and closely located to ependymal cells while others were more elongated and positioned tangentially to the ependymal layer (Figures 5A–C, arrows). Analysis of GFAP identified the subventricular astrocyte band that was located immediately below the ependymal cells (Figure 5D). Some cells had processes inserted among the ependymal cells, directly contacting the CSF (Figures 5E,F, inset and arrows). SVCT2 expression was identified in the ependymal cells and in the SVZ. Whereas the immunoreaction of the ependymal cells was intense throughout the whole cell, the reaction to SVCT2 was concentrated in specific areas of the cells in the SVZ (Figures 5G,J). The signal for SVCT2 rarely co-localized with GFAP-positive or βIII-tubulin-positive cells (Figures 5I,L).

### Vitamin C increases SVCT2 and βIII-tubulin expression

**Figure 6:**
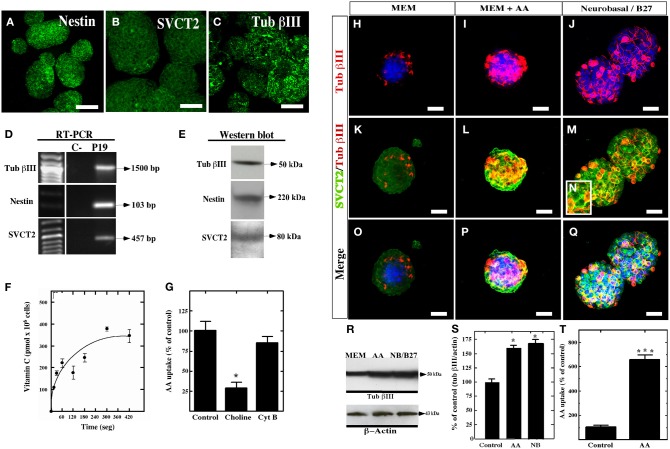
**Expression and function of SVCT2 in P19 cells. (A–C)** Culture of P19 cells for 4 days *in vitro* in MEM medium. The expression of nestin **(A)**, SVCT2 **(B)**, and βIII-tubulin **(C)** was analyzed in the resultant neurospheres. **(D)** RT-PCR analysis using specific primers for βIII-tubulin, nestin, and SVCT2. **(E) 3** Western blot analysis using antibodies that recognize βIII-tubulin, nestin, and SVCT2. **(F)** Functional analysis of the uptake of 100 μM AA over 7 min. **(G)** Uptake of 100 μM AA in the presence/absence of sodium ions or cytochalasin **(B)**. **(H–Q)** Analysis with anti-SVCT2 and anti-βIII-tubulin antibodies in P19 cell cultures at 4 days *in vitro* using different culture conditions. Topro was used to identify the nucleus of the cells (blue). **(H,K,O)** Cells cultured in MEM medium. **(I,L,P)** Cells cultured in MEM medium supplemented with 400 μM vitamin C. **(J,M,Q)** Cells cultured in neurobasal/B27. **(R)** Western blot analysis for βIII-tubulin in P19 cells cultured in the presence of AA or Neurobasal/B27. **(S)** Quantitative analysis of the Western blot analysis. **(T)** Uptake of 100 μM AA in P19 cells cultured in MEM supplemented with 400 μM of vitamin C for 4 days *in vitro*. The data represent the average and standard deviation of three experiments analyzed by One-Way ANOVA with the Bonferroni post-test. ^*^p < 0.05, ^***^p < 0.005. Scale bars in **(A–C)**, 50 μm, **(H–Q)**, 30 μm.

Because various factors can enhance neural differentiation (Dhara and Stice, 2008), the effect of vitamin C on neural differentiation was determined using P19 cells. P19 cells form neurospheres *in vitro*, which are immunoreactive to nestin, SVCT2 and βIII-tubulin (Figures 6A–C). The expression of these markers was also confirmed using RT-PCR (Figure 6D) and Western blot analyses (Figure 6E). Moreover, functional studies revealed that AA is incorporated at a velocity of 100 pmoles × 10^6^ cells/min in the P19 cells, an uptake that remained constant for up to two min of transport (Figure 6F). In the absence of sodium, AA uptake was inhibited by 70% (Figure 6G). To confirm that AA is not being transported by GLUTs, AA transport was determined in the presence of cytochalasin B, an inhibitor of GLUT transporters that incorporates DHA. The presence of this molecule did not inhibit AA transport (Figure 6G). Consequently, these results indicate that P19 cells express functional SVCT2.

To evaluate the effect of vitamin C on neurosphere formation by P19 cells, the culture were maintained with MEM (Figure 6H,K,O) or supplemented with 200 or 400 μM AA for four days (Figures 6I,L,P). As a positive control, the neurospheres were cultured in neurobasal/B27 medium to stimulate neural differentiation (Figures 6J,M,N,Q). After culture in MEM alone, the neurospheres had a lower number of βIII-tubulin-positive cells, even when the majority of the cells in the neurosphere were slightly positive for SVCT2 (Figure 6K). After treating the neurospheres with 400 μM AA vitamin C, an increase in neural differentiation was detected as observed by the presence of intensely βIII-tubulin-positive cells (Figure 6I). These cells were also highly immunoreactive to SVCT2 (Figures 6L,P). A similar response was observed with neurospheres cultured with neurobasal/B27 medium (Figures 6J,M,Q). Western blot analysis confirmed that AA as well as neurobasal/B27 medium increased the levels of βIII-tubulin by two-fold (Figures 6R,S). Finally, AA-treated P19 cells increased AA incorporation by approximately six-fold as compared to the control (Figure 6T).

**Figure 7:**
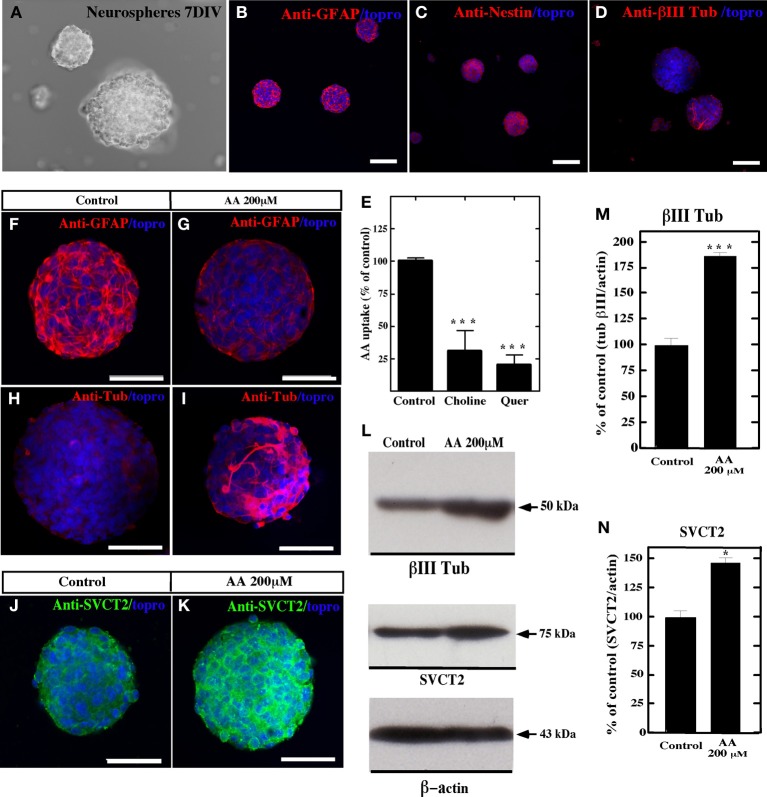
**Vitamin C treatment increases neural differentiation in neurospheres**. *In vitro* culture of neurospheres at 7 days using different culture conditions, and analysis with anti-GFAP, anti-nestin, anti-SVCT2 and anti-βIII-tubulin. Topro was used to identify the nucleus of the cells (blue). **(A–D)** Neurospheres cultured in NeuroCult NS-A medium showed immunoreaction for anti-GFAP and anti-nestin. Low immunoreaction for anti-βIII-tubulin was observed. **(E)** Uptake of 100 μM AA (10 min) and effect of choline and quercetin in cultured neurospheres. (**F–K)** Neurospheres cultured in NeuroCult NS-A medium supplemented with 200 μM vitamin C **(G,I,K)** or control neurospheres without supplementation **(F,H,J)**. Increased βIII-tubulin-positive cells were detected in AA-supplemented neurospheres **(I)**. **(L)** Western blot analysis for βIII-tubulin and SVCT2 expression in neurospheres cultured in the presence of AA or control medium. (**M,N)** Quantitative analysis of the Western blot. The data represent the average and standard deviation of three experiments analyzed by One-Way ANOVA with the Bonferroni post-test. ^*^p < 0.05, ^***^p < 0.005. Scale bars in **(A–D)**, 120 μm; **(F–K)**, 50 μm.

Similar results were observed using primary neurospheres isolated from the lateral wall of the lateral ventricle of adult rats supplemented with 200 μM AA or 400 μM (data not shown) for four days. After 7 days *in vitro* (Figure 7A), the neurospheres cells showed positive immunostaining for anti-GFAP and anti-nestin (Figures 7B,C); however, low immunoreaction was observed with anti-βIII-tubulin (Figure 7D). In addition, uptake of AA was inhibited by choline and quercetin (Figure 7E). When the cells were treated with AA, decreased anti-GFAP immunoreaction was observed (Figures 7F,G); however, the reaction for βIII-tubulin increased (Figures 7H,I,L,M). Additionally, an increased immunoreaction for SVCT2 was observed by immunohistochemistry and Western blot analyses (Figures 7J,K,L,N).

## Discussion

Different studies suggest that intracellular vitamin C may regulate the activation of diverse protein kinases, and thereby regulate transcription factor activity and the expression of pro-neural genes (Bowie and O'Neill, 2000; Carcamo et al., 2002; Park et al., 2005; Frebel and Wiese, 2006; Mimori et al., 2007). Vitamin C also helps to maintain iPS cells *in vitro*, which favored their proliferation (Esteban et al., 2010). Neural tissue has been shown to attain AA concentrations that rank among the highest of mammalian tissues (Horning et al., 1975; Kratzing et al., 1982; Milby et al., 1982). In fact, vitamin C levels are particularly high in fetal rat brain, doubling from the 15th to the 20th day of gestation.

**Figure 8:**
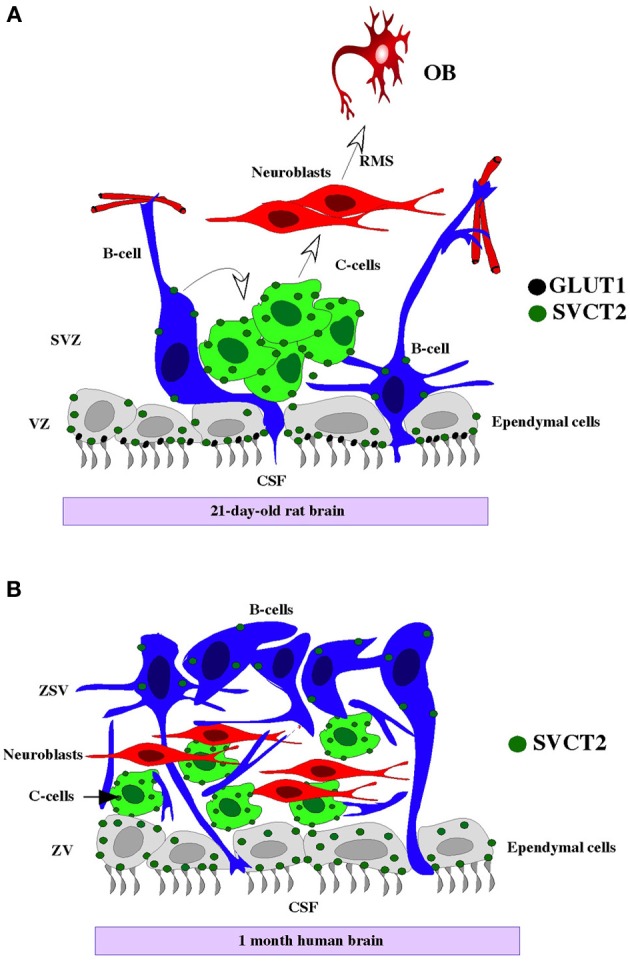
**Cytoarchitecture of the human and rat neurogenic niche and SVCT2 expression**. Rat **(A)** and human neurogenic niche **(B)**, showing type-B GFAP-positive cells (blue cells) in the ependymal, contacting the CSF (grey cells) and subventricular neuroblasts (red cells). SVCT2 is expressed principally in the ependyma and in GFAP-negative and βIII-tubulin-negative type C subventricular progenitor cells (green cell). OB, olfactory bulb; CSF, cerebrospinal fluid; RMS, rostral migratory stream; VZ, ventricular zone; SVZ, subventricular zone.

To determine the postnatal period during which the neurogenic niche is formed, a detailed immunochemical characterization of the first 3 weeks of postnatal development was conducted. Cellular distribution during this period was compared to the cellular distribution of the neurogenic niche and the RMS of the adult brain. Ependymal differentiation was observed beginning at day 7 postnatal development (data not shown), which was similar previous studies (Silva-Alvarez et al., 2005; Spassky et al., 2005). Between days 7 and 21 of development, an intense immunoreactivity to GLUT1 (Silva-Alvarez et al., 2005) and the S100 protein was observed (Figure 8A). However, the radial glia was not present (Hartfuss et al., 2001). Thus, the first cell in the neurogenic niche to differentiate itself was the ependymal cell. Ependymal differentiation could be directly related to the subsequent formation of the neurogenic niche. Noggin secretion by the ependyma stimulates the neurogenic process, acting as an antagonist of bone morphogenic proteins 2 and 4 (BMP2 and BMP4, respectively), factors that stimulate gliogenesis (Lim et al., 2000; Sabo et al., 2009).

GFAP-positive cells were only identified in the SVZ at day 15, indicating the presence of type-B precursor cells (Doetsch et al., 1999; Sanai et al., 2011). The distribution of these cells was similar to what has been described previously for the adult brain (Doetsch et al., 1999; Ma et al., 2005, 2009; Nualart et al., 2012). In addition to the type-B cells, neuroblasts that form the RMS were also identified. Furthermore, the RMS was composed preferentially of cells from the neural lineage reactive to βIII-tubulin and GFAP (Alvarez-Buylla and Lim, 2004; Merkle et al., 2007). The formation of the RMS is additional evidence for concluding that the neurogenic niche forms at day 15. Using *in vivo* BrdU labeling, proliferative type-B cells increased notably at day 21 (Doetsch and Alvarez-Buylla, 1996; Doetsch et al., 1997; Kee et al., 2002) and were in close contact with the ependymal layer. These cells had a long process that contacted the blood vessels forming a specialized vascular niche (Voigt, 1989; Leprince and Chanas-Sacre, 2001; Alvarez-Buylla and Garcia-Verdugo, 2002; Noctor et al., 2002; Mirzadeh et al., 2008; Shen et al., 2008; Tavazoie et al., 2008). Therefore, factors arising from the CSF and blood vessels could regulate the differentiation of the neurogenic niche (Shen et al., 2004; Mirzadeh et al., 2008).

Previous studies have observed SVCT2 expression in cerebral cortex, hypothalamus, hippocampus, thalamus and cerebellar neurons (Tsukaguchi et al., 1999; Castro et al., 2001; Astuya et al., 2005; García et al., 2005; Caprile et al., 2009; Nualart et al., 2012). This transporter is fundamental for the neural development of CNS, which was confirmed with the use of SVCT2 knock-out animals (Sotiriou et al., 2002). Qiu et al. (2007) demonstrated that neural cultures from SVCT2 knock-out animals had lower rates of neurite growth and reduced neural activity (Qiu et al., 2007). SVCT2 expression was also reported in hypothalamic tanycytes, ependymal cells, marginal astrocytes and microglia (García et al., 2005; Mun et al., 2006; Nualart et al., 2012).

Vitamin C may induce neural and glial differentiation in embryonic cortical precursors and mesencephalic precursor cells (Lee et al., 2000, 2003; Yan et al., 2001). AA concentrations in the CSF may reach up to 500 and 200–400 μM in regions of the cerebral parenchyma (Spector and Lorenzo, 1974). The presence of the vitamin C transporter, SVCT2, in the ventricular neurogenic area after 15 days postnatal development was analyzed in the present study. The expression of SVCT2 in ventricular and subventricular areas has only been described in the embryonic rat brain (Caprile et al., 2009). However, the expression of SVCT2 in postnatal neurogenic areas remains unknown. Using immunohistochemical analysis, the present study was the first to identify SVCT2 expression in postnatal rat brain and in the human neurogenic niche at 1 month postnatal development (Figures 8A,B). These results were confirmed by *in situ* hybridization, observing positive hybridization in the SVZ and RMS. Double and triple-labeling techniques revealed low co-localization of SVCT2 (rat and human) with GFAP and βIII-tubulin, indicating that type-B cells and neuroblasts would have a low capacity to transport vitamin C. Alternatively, co-localization of SVCT2 with BrdU-positive cells, in particular in the SVZ, suggests that type-C cells with high mitotic activity (Doetsch et al., 1999; Alvarez-Buylla and Garcia-Verdugo, 2002; Kriegstein and Alvarez-Buylla, 2009) preferentially express this transporter. SVCT2 also co-localized with BrdU in the RMS, suggesting the presence of progenitors or similar cells (Doetsch and Alvarez-Buylla, 1996).

The transport of vitamin C to type-C cells of the SVZ can be explained by diffusion from the CSF as vitamin C is present at concentrations up to 10-fold higher than that observed in blood (Tsukaguchi et al., 1999; García et al., 2005). Finally, as observed in the present study, SVCT2 was widely expressed in the postnatal neurogenic area and RMS, suggesting that mitotically active type-C progenitors preferentially express the transporter. Unfortunately, it was not possible to co-localize SVCT2 with markers of type C cells due to technical problems associated with the use of picric acid within the Bouin solution that was used as a fixative for identifying SVCT2.

Precursor cells have the ability to form neurospheres (Reynolds and Weiss, 1992, 1996; Doetsch et al., 1999; Nunes et al., 2003; Chojnacki et al., 2009). In this work, primary neurospheres from rat brain and P19-derived neurospheres expressed nestin, GFAP and βIII-tubulin, and functional SVCT2. Supplementation of 4 days *in vitro* P19-derived neurospheres with 400 μM vitamin C increased their expression of βIII-tubulin, which has also been observed in embryonic precursor cells (Yan et al., 2001). In addition, a six-fold increase in the incorporation of AA was observed in P19 cells cultured with vitamin C, which was similar to the results obtained after culturing cells in Neurobasal/B27 medium, a condition that induces neural differentiation (Hemmati et al., 2003; Beier et al., 2007). Finally, we used primary neurospheres isolated from rat brain to define the effect of 200 or 400 μM vitamin C; 200 μM vitamin C produced a similar differentiation effect to that observed in P19 cells treated with 400 μM vitamin C. The differential response to vitamin C concentration may be due to the level of SVCT2 expression and/or its functional activity in the cells. Because vitamin C uptake by cells is dependent on the affinity of the transporter as well as its concentration in the cellular membrane, we think that the functional activity of SVCT2 is increased in neurospheres. Additionally, increased SVCT2 expression was observed in primary neurospheres after AA treatment. In conclusion, we suggest that vitamin C and SVCT2 may be important in inducing neurogenesis in postnatal stages.

The specific mechanisms through which vitamin C and SVCT2 induce neural differentiation remain unknown although some evidence suggests the involvement of gene expression related to neurogenesis and maturation (Lee et al., 2003; Shin et al., 2004; Yu et al., 2004). SVCT2 may be regulated in differentiation through its phosphorylation sites dependent on protein kinase C (PKC) and protein kinase A (PKA) (Daruwala et al., 1999; Rajan et al., 1999; Tsukaguchi et al., 1999; Wang et al., 1999, 2000) since its phosphorylation is essential for translocation to the cellular membrane and subsequent AA uptake (Wu et al., 2007). The level of SVCT2 in the cellular membrane is also increased by prostaglandin E through the activation of the EP4 and PK prostaglandin receptor (Wu et al., 2007).

In this study, differentiation of the postnatal neurogenic niche at days 15 and 21 was analyzed by determining the expression pattern of the AA transporter, SVCT2 (Figure 8). SVCT2 was expressed in the highly proliferative cells of the neurogenic niche. Additionally, primary neurospheres prepared from rat brain and the P19 teratocarcinoma cell lines also expressed SVCT2, which is involved in AA uptake. Vitamin C-induced neural differentiation increased βIII-tubulin and SVCT2 expression.

### Conflict of interest statement

The authors declare that the research was conducted in the absence of any commercial or financial relationships that could be construed as a potential conflict of interest.
